# Burnout in mental health services in Ireland during the COVID-19 pandemic

**DOI:** 10.1192/bjo.2023.552

**Published:** 2023-10-06

**Authors:** Dimitrios Adamis, Elisha Minihan, Noel Hannan, Anne M. Doherty, Fiona McNicholas

**Affiliations:** Department of Psychiatry, Sligo Mental Health Services, Ireland; and School of Medicine and Medical Science, University College Dublin, Ireland; School of Medicine and Medical Science, University College Dublin, Ireland; Department of Psychiatry, Saint John of God – Liffey Region Services, Ireland; and Department of Psychiatry, Saint John of God – Dublin South East Services, Ireland; Department of Psychiatry, Mater Misericordiae University Hospital, Ireland; School of Medicine and Medical Science, University College Dublin, Ireland; Department of Psychiatry, Children's Health Ireland (CHI) at Crumlin, Our Lady's Children's Hospital, Ireland; and Department of Psychiatry, Lucena Clinic Services, Ireland

**Keywords:** Burnout, mental health services, COVID-19, Ireland, occupational stress

## Abstract

**Background:**

Burnout is a consequence of chronic occupational stress. Specific work-related factors may contribute to burnout experienced by those working in mental health services (MHS), many of which have increased since the COVID-19 pandemic.

**Aims:**

To examine personal, work- and patient-related burnout among MHS staff in Ireland during the COVID-19 pandemic, and explore the impact of work-related conditions on burnout.

**Method:**

We conducted a cross-sectional survey of three MHS across Ireland utilising a study-specific questionnaire, the Copenhagen Burnout Inventory and the Effort–Reward Imbalance scale.

**Results:**

Of 396 participants, 270 (70.6%) were female. Moderate and high personal burnout was experienced by 244 (64.1%) participants; work-related burnout by 231 (58.5%) participants and patient-related burnout by 83 (21.5%) participants. Risk factors for both personal and work-related burnout were female gender, urban service, time spent outside main responsibilities, overcommitment, high score on the Effort–Reward Imbalance scale and intention to change job. Being younger, with high workload and deterioration of personal mental health during the pandemic was associated with higher personal burnout, whereas a lack of opportunity to talk about work-related stress contributed to work-related burnout. Fewer factors were associated with patient-related burnout, namely overcommitment, working in urban services and poorer physical and mental health during the COVID-19 pandemic.

**Conclusions:**

High levels of personal and work-related burnout were found among mental health workers. The weak association with COVID-19-related factors suggest levels of burnout predated the pandemic. This has implications for MHS given the recognised additional work burden created by COVID-19.

High levels of occupational stress among healthcare staff have implications for patient safety and service delivery.^1^ In Ireland, doctors working in hospitals have high rates of occupational stress, which are higher than doctors working elsewhere in Europe.^2^ Those working in the speciality of psychiatry may be particularly vulnerable to developing burnout, as evidenced by higher levels of occupational stress reported than for other specialities^3^ and as a result of being exposed to, and expected to manage, stress from numerous sources.^4^ Although most of this research was conducted before the COVID-19 pandemic, it was expected that the COVID-19 pandemic would increase psychological distress and contribute to deterioration in pre-existing mental health conditions, as well as increase new presentations. Such increases in mental health distress have both direct and indirect effects for mental health service (MHS) providers. The psychological footprint of a pandemic is recognised to be far greater and longer lasting than the medical one, contributing to additional demands on MHS. At a time when clinical services were already overstretched, the COVID-19 pandemic is likely to compound this.^5^ Unique COVID-19-related stressors (at least in hospital settings) included fears of adequate access to personal protective equipment (PPE); the risk of personal infection; transmission of infection to patients, colleagues or family; balancing duty of care to family at home and continuing to provide a service despite these demands.^6^ However, although these additional stressors have been investigated widely in general hospitals (COVID-19 wards, critical care units) and accident and emergency departments, few studies have examined such stressors within a community MHS,^7^ with some initial justification on the basis that community services were not front-line and were therefore somewhat protected from the psychological distress typically expected among front-line workers, and that adaptations to delivery (e.g. remote delivery) were easier to implement.^7^ Subsequent work has challenged this assumption, recognising that working remotely carries its own additional stress.^8^

In that context, this study was designed to examine the level of burnout in staff working in MHS across Ireland during the COVID-19 pandemic. We wanted to examine the level of personal, work- and patient-related stress; to explore and understand the impact of the COVID-19 pandemic and the consecutive restrictions imposed on burnout; and to examine specific work-related conditions on burnout levels.

## Method

This was a cross-sectional survey examining burnout among clinicians in MHS over the time period December 2021 to March 2022. Three MHS in Ireland were chosen, and for reasons of confidentiality these have been named A, B and C; two (A and C) were in urban areas and one was in a semi-rural area of Ireland (B). All staff working in these three MHS were eligible for participation, with no exclusion criteria applied.

### Questionnaires/scales

A study-specific questionnaire previously developed and used in other studies on burnout was used,^4,6,9^ with minor adaptations. It included basic question on demographics, work-related conditions and specific questions relating to the pandemic, using yes/no or Likert scale responses (see Tables 1 and 2). The Copenhagen Burnout Inventory (CBI) was the main outcome scale, examining levels of personal stress or burnout, along with the degree of burnout perceived to be related to work and client/patient population.^10^ It has three domains: work-related burnout (seven items), personal burnout (six items) and patient-related burnout (six items). All 19 items are scored with a five-point Likert scale: always/very high degree, often/high degree, sometimes/somewhat, seldom/low degree and never/almost never (scored 4, 3, 2, 1 and 0, respectively). Maximum scores for subscales are 24 for both personal and patient-related burnout, and 28 for work-related burnout (total scale score maximum 76). Higher scores indicate higher levels of burnout. It is well-validated with a high reliability/agreement.^11^ The Effort–Reward Imbalance (ERI) scale (short version) was also used. This includes 16 items divided in three domains measuring effort (three items), reward (seven items) and overcommitment (six items).^12^ Higher scores indicate more effort, less reward and overcommitment. Each item is rated on a four-point Likert scale. The ERI has good psychometric properties, with alpha coefficients of 0.85 for effort and 0.84 for reward.^13^ It has previously been used and validated, with good correlation between high effort, low reward and poor health. The scale allows for the calculation of an effort/reward ratio (ERIdx), which can be log10 transformed and used as a continuous variable. An unfavourable ERIdx score of >1 indicates more effort for each reward.

**Table 1:** Descriptive statistics for work-related questions

	*n*	%
Area you work in		
Clinical	289	74.5
Non-clinical	99	25.5
Total	388	100.0
How much of your time do you think you spend on what you think is outside your area of responsibility/expertise?		
A little	151	38.6
A moderate amount	175	44.8
A lot of my time	65	16.6
Do you feel valued in your job?		
No	143	36.4
Not sure	76	19.3
Yes	174	44.3
Total	393	100.0
Do you feel staff in your service experience good job satisfaction?		
No	179	45.5
Yes	118	30.0
Not sure	96	24.4
Total	393	100.0
Did you have any stress reduction training in your professional/job training?		
No	284	71.9
Not sure	34	8.6
Yes	77	19.5
Total	395	100.0
Do you feel your organisation has tried to reduce work-related stress?		
No	269	68.8
Yes	70	17.9
Not sure	52	13.3
Total	391	100.0
Did you have any stress reduction training in your job before COVID-19?		
No	306	78.3
Yes	56	14.3
Not sure	29	7.4
Total	391	100.0
Did you have any stress reduction training in your job after COVID-19?		
No	310	79.3
Yes	47	12.0
Not sure	34	8.7
Total	391	100.0
Do you have anyone in your organisation you can talk to about work-related stress or burnout?		
No	134	34.0
Yes	205	52.0
Not sure	55	14.0
Total	394	100.0
How would you rate your current work ability compared with your lifetime best?		
Better	77	20.3
Worse	180	47.5
Same	122	32.2
Total	379	100.0
There were occasions when I think I should have taken time off for illness but did not		
Yes	262	68.2
No	122	31.8
Total	384	100.0
Have you seriously thought of changing jobs in the past 6–12 months?		
No	137	34.7
Not sure	14	3.5
Yes	244	61.8
Total	395	100.0

**Table 2:** Descriptive statistics for COVID-19-related questions

	*n*	%
Was there a change in your overall workload during COVID-19?		
Increased	308	82.6
Same	43	11.5
Decreased	22	5.9
Total	373	100.0
Was there any change in staffing levels in your department during COVID-19?		
Increased	35	9.3
Same	139	37.1
Decreased	201	53.6
Total	375	100.0
Was there any change in level of clinical support in your department during COVID-19?		
Increased	31	8.3
Same	184	49.1
Decreased	160	42.7
Total	375	100.0
Was there any change in the level of administrative support in your department during COVID-19?		
Increased	31	8.3
Same	189	50.4
Decreased	155	41.3
Total	375	100.0
Relative to usual activity, do you think patient contact/referrals changed during COVID-19?		
Increased	213	57.9
Same	67	18.2
Decreased	88	23.9
Total	368	100.0
Relative to your usual satisfaction with your work, has this changed during COVID-19?		
Increased	47	12.7
Same	136	36.7
Decreased	188	50.7
Total	371	100.0
If required, do you feel you had adequate access to PPE during COVID-19?		
Yes	332	90.0
No	37	10.0
Total	369	100.0
Did you feel you were provided with adequate information on COVID-19 from your organisation/department?		
Yes	337	91.1
No	33	8.9
Total	370	100.0
Was there any change in your physical health in relation to/during COVID-19?		
Improved	12	3.3
No change	217	59.5
Deteriorated	136	37.3
Total	365	100.0
Was there any change in your mental health in relation to/during COVID-19?		
Improved	10	2.7
No change	152	41.4
Deteriorated	205	55.9
Total	367	100.0
Was there any change in the physical health of a family member linked to/during COVID-19?		
Improved	4	1.1
No change	236	65.9
Deteriorated	118	33.0
Total	358	100.0
Was there any change in the mental health of a family member linked to/during COVID-19?		
Improved	3	0.8
No change	184	51.8
Deteriorated	168	47.3
Total	355	100.0
Was there any change to your eating habits during COVID-19?		
Improved	42	11.3
No change	177	47.7
Deteriorated	152	41.0
Total	371	100.0
Did your relationship with alcohol improve or disimprove during COVID-19?		
Improved	44	12.1
No change	232	63.6
Deteriorated	89	24.4
Total	365	100.0
Was there any change to your sleeping habits during COVID-19?		
Improved	11	3.0
No change	168	45.7
Deteriorated	189	51.4
Total	368	100.0
Was there any change to your exercise habits during COVID-19?		
Improved	119	32.2
No change	113	30.6
Deteriorated	137	37.1
Total	369	100.0
How likely is it that any behavioural change you have made during COVID-19 will continue post pandemic?		
Likely	222	60.5
Unlikely	24	6.5
Not sure	121	33.0
Total	367	100.0
How confident did you feel about access to and correct use of PPE?		
Confident	327	88.6
No confident	42	11.4
Total	369	100.0
How aware are you about the occupational supports available to you through your workplace?		
Very high degree of awareness	32	8.8
High degree of awareness	68	18.7
Somewhat aware	151	41.6
Low degree of awareness	74	20.4
Very low degree of awareness	38	10.5
Total	363	100.0
How likely are you to access these services if you needed assistance?		
Likely	139	38.3
Unlikely	92	25.3
Not sure	132	36.4
Total	363	100.0

PPE, personal protective equipment.

Procedure: all staff working in the three MHS were invited to participate, using a mixed-method approach to maximise participant reach. Invitations were disseminated by email, sent from administration and containing a link to the survey and a study information sheet, with a reminder email sent 4 weeks later (the study was offered twice). Hard copies were also left in the workplaces with a prepaid stamped envelope addressed to investigators, and the survey was mentioned at team meetings. The end of the study was declared after 6 weeks of the reminders.

The study received local research ethics committee approval at each site (Sligo University Hospital number 867 21/4/21; Saint John of God identifier 779 on May 2021; and University College Dublin Research Ethics Committee reference LS-19-103-Minihan-McNicholas on January 2021). Written consent was obtained anonymously by ticking the relevant box in the information for participants or selecting the consent box in the online version. The participation was strictly voluntary and no reward was offered.

### Statistical analysis

Categorical variables were summarised as counts and percentages, and continuous variables were summarised as means and s.d. If the continuous variable was not normally distributed, the median, interquartile range, and minimum and maximum were reported. Division of CBI total scale and subscales into categories was based on the distribution of scores. Respondents scoring below average (50% of sample scores) were characterised as experiencing ‘low/no’ burnout, the 25% scoring above average were characterised as ‘moderate’ burnout and the top 25% were characterised as ‘high’ burnout, consistent with previous CBI reporting methodology.^6,11^ For bivariate analysis, parametric or non-parametric tests were used as appropriate to data distribution. To examine the main effects of individual variables on the three subscales of CBI, a multivariate analysis was used. To avoid multicollinearity and reduce the number of independent variables, those with high correlations were not included in the initial model. Variables that did not significantly contribute to the model were dropped individually until a parsimonious model was achieved. All analysis was done with IBM SPSS version 25 software.

## Results

A total of 1701 staff were eligible to participate in the study, and 475 responded (response rate 27.93%). Response rates varied by service: 18.1% (247/1368) for MHS A, 62.43% (108/173) for MHS B and 75% (120/160) for MHS C. A further 79 were excluded because of significant questionnaire incompletion, reducing the final number analysed to *N* = 396.

### Descriptive statistics

Of the total 396 participants, 279 were female (70.5%). The mean age of participants was 44.24 years (s.d. = 10.97). Most respondents were clinicians (*n* = 289, 74.5%), followed by staff in support services (*n* = 57, 14.7%) and administrative/secretarial roles (*n* = 42, 10.8%), such that the non-clinical group comprised 99 respondents (25.5%). The median duration of time spent working in the service was 15 years (interquartile range 18, range 1–43). Most respondents worked predominantly in intellectual disability MHS (*n* = 116, 39.3%), followed by adult MHS (*n* = 95, 32.2%), child and adolescent MHS (*n* = 58, 19.7%) and old-age MHS (*n* = 9, 3.1%). Others included supporting (maintenance) services for all groups (*n* = 17, 5.8%). Work-related descriptive statistics are presented in Table 1, and COVID-19-related questions are shown in Table 2.

Descriptive statistics for the CBI are presented as continuous variables, using cut-off points (Table 3), along with the mean ERI scores. Most participants scored in the moderate and high level of work-related and personal burnout, with corresponding mean scores of 52.71 (s.d. = 20.6) and 53.55 (s.d. = 20.41). Lower mean scores (31.35, s.d. = 19.97) were present for patient-related burnout.

**Table 3:** Copenhagen Burnout Inventory and Effort–Reward Imbalance scale

	*n*	%	Mean	s.d.
Work-related burnout				
No/low	165	41.7	52.71	20.61
Moderate	159	40.2		
High	72	18.2		
Total	396	100.0		
Personal burnout				
No/low	152	38.4	53.55	20.41
Moderate	177	44.7		
High	67	16.9		
Total	396	100.0		
Patient-related burnout				
No/low	303	78.5	31.35	19.97
Moderate	71	18.4		
High	12	3.1		
Total	386	100.0		
Total burnout				
No/low	221	55.8	46.35	17.26
Moderate	155	39.1		
High	20	5.1		
Total	396	100.0		
Total effort			9.1	1.96
Total reward			17.36	3.66
Total overcommitment			14.57	3.69
Effort–reward index (ERIdx)			1.31	0.49

### Bivariate statistics

Examination of levels of burnout between genders showed that women were significantly more likely to have higher total, personal and work-related burnout compared with men (total: *t* = −3.159, d.f. = 393, *P* = 0.002; personal: *t* = −3.567, d.f. = 393, *P* < 0.001; work-related: *t* = −3.784, d.f. = 393, *P* < 0.001). No gender-specific difference was found for patient-related burnout (*t* = −0.422, d.f. = 383, *P* = 0.673).The age of participants was not significantly correlated with burnout (total, personal, work-related and patient-related), but years working in the service was positively corelated with total burnout (*r* = 0.161, *P* = 0.025), personal burnout (*r* = 0.181, *P* = 0.011) and work-related burnout (*r* = 0.186, *P* = 0.009). No such findings existed for patient-related burnout (*r* = 0.035, *P* = 0.632). In addition, log-ERIdx (higher scores indicate more effort with less reward) was positively corelated with total burnout (*r* = 0.537, *P* < 0.001) and all subscales (personal burnout: *r* = 0.483, *P* < 0.001; work-related burnout: *r* = 0.584, *P* < 0.001; patient-related burnout: *r* = 0.282, *P* < 0.001). No significant differences were found between clinical and non-clinical staff in terms of levels of burnout (total, personal, work-related, patient-related), overcommitment and log-ERIdx.

### Multivariate analysis

To control for confounders, a multivariable model was constructed with three dependent variables (work-related burnout, personal burnout, patient-related burnout) and a number of independent variables (in the initial model): (a) demographics (gender, age, years of working in MHS, location), (b) work-related questions (time spent outside of area of responsibility/expertise, clinical/non clinical work, intention to change job, stress reduction training, availability to discuss burnout, current work compared with previous work) and (c) COVID-19-related questions (workload during COVID-19, job satisfaction during COVID-19, personal and family health during COVID-19, access and correct use of PPE, and awareness of occupational support in the workplace). In addition, the log-ERIdx (effort–reward imbalance) and total overcommitment were added as independent variables. The final parsimonious model is presented in Table 4 (tests of between-participant effects) and Table 5 (parameter estimates). The final model did not depart from the assumption of equality of covariance matrices (Box's M test, mean 18.403, F = 1.032, d.f.1 = 12, d.f.2 = 859.781, *P* = 0.417) and the assumption of homogeneity of variance (Levene's test; personal burnout: F = 1.421, d.f.1 = 272, d.f.2 = 44, *P* = 0.080; work-related burnout: F = 1.192, d.f.1 = 272, d.f.2 = 44, *P* = 0.245; patient-related burnout: F = 1.010, d.f.1 = 272, d.f.2 = 44, *P* = 0.505).

**Table 4:** Multivariate analysis, tests of between-participant effects

Source	Type 3 sum of squares	d.f.	Mean square	F	Significance
Intercept					
Personal burnout	3553.361	1	3553.361	20.126	<0.001
Work-related burnout	3356.182	1	3356.182	24.081	<0.001
Patient-related burnout	50.664	1	50.664	0.175	0.676
Mental health service (locality)					
Personal burnout	3963.675	2	1981.837	11.225	<0.001
Work-related burnout	3585.864	2	1792.932	12.865	<0.001
Patient-related burnout	2108.729	2	1054.365	3.643	0.027
Gender					
Personal burnout	1844.424	1	1844.424	10.447	0.003
Work-related burnout	1706.830	1	1706.830	12.247	0.001
Patient-related burnout	613.506	1	613.506	2.120	0.146
Time outside responsibilities					
Personal burnout	2624.145	2	1312.072	7.432	0.003
Work-related burnout	3018.680	2	1509.340	10.830	<0.001
Patient-related burnout	79.858	2	39.929	0.138	0.871
Changing job					
Personal burnout	2693.402	2	1346.701	7.628	0.001
Work-related burnout	2865.306	2	1432.653	10.279	<0.001
Patient-related burnout	879.557	2	439.779	1.520	0.220
Anyone in your organisation that you can talk to for burnout					
Personal burnout	230.703	2	115.351	0.653	0.521
Work-related burnout	1609.141	2	804.571	5.773	0.003
Patient-related burnout	1762.667	2	881.333	3.046	0.049
Overall workload during COVID-19					
Personal burnout	787.170	2	393.585	2.229	0.109
Work-related burnout	20.564	2	10.282	0.074	0.929
Patient-related burnout	1288.690	2	644.345	2.227	0.110
Change in job satisfaction during COVID-19					
Personal burnout	747.756	2	373.878	2.118	0.122
Work-related burnout	1083.934	2	541.967	3.889	0.022
Patient-related burnout	402.170	2	201.085	0.695	0.500
Personal mental health					
Personal burnout	1447.613	2	723.807	4.100	0.018
Work-related burnout	1867.270	2	933.635	6.699	0.001
Patient-related burnout	4253.318	2	2126.659	7.349	0.001
Family member mental health					
Personal burnout	638.994	2	319.497	1.810	0.166
Work-related burnout	749.003	2	374.501	2.687	0.070
Patient-related burnout	1678.249	2	839.124	2.900	0.057
Personal physical health					
Personal burnout	854.001	2	427.000	2.419	0.091
Work-related burnout	477.370	2	238.685	1.713	0.182
Patient-related burnout	3346.107	2	1673.054	5.781	0.003
Age					
Personal burnout	759.243	1	759.243	4.300	0.039
Work-related burnout	385.599	1	385.599	2.767	0.097
Patient-related burnout	43.232	1	43.232	0.149	0.699
log-ERIdx					
Personal burnout	1399.393	1	1399.393	7.926	0.005
Work-related burnout	3568.957	1	3568.957	25.608	<0.001
Patient-related burnout	158.995	1	158.995	0.549	0.459
Overcommitment					
Personal burnout	9620.546	1	9620.546	54.490	<0.001
Work-related burnout	7962.080	1	7962.080	57.129	<0.001
Patient-related burnout	9076.208	1	9076.208	31.364	<0.001

ERIdx, effort–reward index.

**Table 5:** Parameter estimates (only significant variables are shown)

					95% Confidence interval	
Dependent variable	B	s.e.	*t*	Significance	Lower bound	Upper bound
Personal burnout						
Intercept	46.819	6.817	6.868	<0.001	33.403	60.235
Service A	−2.896	2.000	−1.448	0.149	−6.833	1.041
Service B	−10.292	2.272	−4.530	<0.001	−14.763	−5.820
Service C	0^a^					
Male	−5.565	1.722	−3.232	0.001	−8.954	−2.176
Female	0^a^					
Time spent on work outside of responsibilities						
A little	−8.378	2.347	−3.569	<0.001	−12.997	−3.759
A moderate amount	−7.702	2.173	−3.544	<0.001	−11.978	−3.425
A lot of my time	0^a^					
Changing job						
No	−5.907	1.887	−3.130	0.002	−9.621	−2.193
Not sure	−11.486	4.028	−2.852	0.005	−19.413	−3.560
Yes	0^a^					
Overall workload						
Increased	5.285	3.316	1.594	0.112	−1.240	11.811
Same	8.173	3.875	2.109	0.036	0.546	15.799
Decreased	0^a^					
Change in job satisfaction						
Increased	−1.440	2.450	−.588	0.557	−6.260	3.381
Same	−4.002	1.951	−2.051	0.041	−7.842	−0.161
Decreased	0^a^					
Personal mental health						
Improved	−6.341	5.419	−1.170	0.243	−17.006	4.324
Same	−5.581	1.984	−2.813	0.005	−9.485	−1.677
Deteriorated	0^a^					
Age	−0.155	0.075	−2.074	0.039	−0.302	−0.008
log-ERIdx	17.153	6.093	2.815	0.005	5.162	29.144
Overcommitment	1.864	0.252	7.382	<0.001	1.367	2.361
Work-related burnout						
Intercept	53.510	6.057	8.835	<0.001	41.590	65.430
Service A	−4.834	1.777	−2.720	0.007	−8.332	−1.336
Service B	−10.230	2.019	−5.068	<0.001	−14.202	−6.257
Service C	0^a^					
Male	−5.353	1.530	−3.500	0.001	−8.364	−2.343
Female	0^a^					
Time spent on work outside of responsibilities						
A little	−8.822	2.085	−4.230	<0.001	−12.926	−4.718
A moderate amount	−8.403	1.931	−4.353	<0.001	−12.203	−4.604
A lot of my time	0^a^					
Changing job						
No	−6.582	1.677	−3.925	<0.001	−9.882	−3.282
Not sure	−10.472	3.578	−2.927	0.004	−17.515	−3.430
Yes	0^a^					
Someone to talk to for burnout						
No	0.592	2.162	0.274	0.784	−3.663	4.847
Yes	−4.441	2.016	−2.203	0.028	−8.408	−0.473
No sure	0^a^					
Change in job satisfaction						
Increased	0.246	2.176	0.113	0.910	−4.037	4.530
Same	−4.426	1.734	−2.553	0.011	−7.838	−1.014
Decreased	0^a^					
Personal mental health						
Improved	−8.313	4.815	−1.727	0.085	−17.789	1.163
Same	−6.238	1.762	−3.540	<0.001	−9.707	−2.770
Deteriorated	0^a^					
log-ERIdx	27.394	5.413	5.060	<0.001	16.740	38.047
Overcommitment	1.696	0.224	7.558	<0.001	1.254	2.137
Patient-related burnout						
Intercept	7.640	8.727	0.875	0.382	−9.536	24.816
Service A	−4.137	2.561	−1.615	0.107	−9.177	0.904
Service B	−7.847	2.909	−2.698	0.007	−13.572	−2.123
Service C	0^a^					
Personal mental health						
Improved	−3.517	6.938	−0.507	0.613	−17.171	10.137
Same	−9.686	2.540	−3.814	<0.001	−14.684	−4.688
Deteriorated	0^a^					
Family mental health						
Improved	−15.706	13.931	−1.127	0.260	−43.122	11.711
Same	4.391	2.186	2.009	0.045	0.089	8.694
Deteriorated	0^a^					
Personal physical health						
Improved	14.511	5.927	2.448	0.015	2.847	26.176
Same	6.733	2.305	2.922	0.004	2.198	11.268
Deteriorated	0^a^					
Overcommitment	1.810	0.323	5.600	<0.001	1.174	2.446

The – /+ sign in front of the estimates shows the direction of the effects in relation to dependent variable(s), e.g. service B had significantly less personal burnout compared with service C. ERIdx, effort/reward ratio.

a. This parameter is set to zero because it is redundant.

The location of the MHS, personal mental health and degree of overcommitment had significant effects in the three subscales of the CBI (Table 4). Gender, time spent outside area of expertise, intention to change job and effort/reward had a significant effect only in work-related and personal burnout (Table 4). Age had only a significant effect in personal burnout. Personal physical health was significantly associated with patient-related burnout. Change in job satisfaction during the COVID-19 pandemic was significantly associated with work-related burnout only. The ability to talk to someone for issues related to burnout was significantly associated with work-related burnout and marginally with patient-related burnout (Table 4). Table 5 presents the effects of each level of the independent variables on the three subscales of the CBI.

After controlling for confounders, it seems that personal burnout is significantly associated with female gender and younger age, work characteristics (service locality), spending a lot of time outside of main responsibilities, higher overcommitment, low reward with more effort and intention to change job (Table 5). Those with the same workload during the COVID-19 pandemic had more personal burnout compared with those with a reduced workload. Job satisfaction remained the same during the pandemic and was related to lower levels of burnout. Those with stable mental health during the pandemic also had lower levels of burnout compared with staff who experienced mental health deterioration.

Similar factors were also related to work-related burnout. These included female gender, overcommitment, high effort with less reward, location of services, spending a lot of time outside of main responsibilities and intention to change job. Reduced work-related burnout was associated with each of the following: availability of someone to talk about work-related stress inside the organisation, stable job satisfaction and stable personal mental health during the pandemic.

Finally, regarding patient-related burnout, demographic variables (age, gender) did not have any significant effect, but overcommitment and location of services did. Personal physical and mental health, and mental health of family members, were significantly associated with patient-related burnout. Those with stable mental health during the COVID-19 pandemic experienced lower levels of burnout, in contrast to the higher levels of burnout found in those with stable or improved physical health during the pandemic.

## Discussion

### Burnout

The majority of those surveyed had moderate to high levels of personal and work-related burnout, as measured by the CBI. This was in stark contrast to the low or absent levels of patient-related burnout, suggesting that despite high overall stress levels, compassion fatigue and empathic distress were low. This concurs with other studies conducted both during the COVID-19 pandemic^6,14^ and before it,^4,15^ and is consistent with meta-analyses and systematic reviews.^3,16^ This finding of relatively high levels of personal and work-related burnout compared with a lower level of patient-related burnout has been found in studies examining other disciplines, e.g. among midwifes^17^ and teachers.^9^ The lower levels of stress and burnout regarding patients (or in the case of teachers, students) suggest that organisational or personal, rather than patient- or student-related factors, are contributory to stress levels. Given the lack of longitudinal data, we are unable to examine whether patient-related stressors changed during the COVID-19 pandemic. It is reassuring that it follows previous patterns of lower levels, indicating that despite anticipated additional patient-related care required during the pandemic, patient-related factors remained low. Burnout is a complex and multifactorial syndrome, making it difficult to attribute its development to a specific cause. It has been suggested that personal burnout may be related to low salary and lack of recognition, whereas work-related burnout is associated with organisational issues such as limited resources and perceived mismanagement resulting in high levels of stress.^17^ It is surprising that no research has explored why patient-related burnout is low compared with other forms of burnout.

### COVID-19-related factors

During the COVID-19 pandemic, most participants (83%) reported increased workload, but this increase did not have any significant effect on burnout. It is possible that the new challenges and the feeling of being active and able to contribute to the well-being of others in this time of uncertainty had a protective effect. For a minority of respondents, where workload either remained the same or reduced, an unchanged level was associated with higher levels of personal burnout. Caution is required, given the small numbers and wide confidence intervals suggesting a type 1 error. Half of the respondents reported reduced job satisfaction during the pandemic, adversely affecting personal and work-related domains of burnout. Those with unchanged job satisfaction reported lower levels of mental health during the pandemic had less burnout in all subscales compared with those who experienced a mental health deterioration. This close association between adverse mental health and burnout has been reported in other studies examining depression and anxiety.^18^ As this study was cross-sectional, a cause–effect relationship cannot be determined. The finding of higher patient-related burnout (typically the last type to increase) in those with unchanged as opposed to deteriorated physical health, might at first glance seem surprising. It is our suggestion that staff who remained physically well continued to present to work, whereas those who became unwell may have taken time off work. As such, the former group may have had to cover gaps in services when other colleagues were on sick leave, and usual annual leave was suspended at that time. In our study, COVID-19-related variables, such as access to PPE or information about COVID-19, did not have a significant effect on burnout, which is at odds with previous studies.^6,9^ This might be accounted for by the timing of our study (conducted toward the end of the second wave of the COVID-19 pandemic, where many of these issues, including uncertainty, had been resolved) and/or of the location of the study (conducted in a community setting as opposed to the hospital setting where access to such protective wear is of greater importance).

Intention to change job and higher time spent outside primary responsibilities were independent predictors of personal and work-related burnout. In contrast, the opportunity to speak to someone inside the organisation about burnout was associated with reduced levels of work-related burnout. The association between intention to change job and burnout is well established.^15,18^ This relationship is reciprocal, and it has been speculated that it may be mediated by organisational factors such as ineffective policies, inadequate workforce planning and recruitment, incompetent implementation planning and ineffective leadership.^18^ Similarly, spending time outside of one's area of responsibilities can generate anxiety as well as a sense of inadequacy or lack of competence, and may be compensated for by increasing additional hours. Being aware about burnout, speaking about it, participating in support groups and increasing social capital in the workplace are protective factors from burnout and reduce the intention to leave.^19^

Our study also unearthed some location specific and unexpected results. Significantly lower rates of all measures of burnout (personal, work-related and patient-related) were found in MHS B, which was located in a semi-rural part of Ireland. In contrast, MHS A and MHS C were large urban areas and had higher burnout rates. This effect was independent of the other examined factors. Tham et al^20^ alluded to the paucity of data examining rural and urban healthcare workers. In their large (*N* = 7846) cross-sectional study conducted in Australia, of whom 18.8% worked in regional or remote areas and 81.2% in metropolitan areas, they reported higher levels of personal accomplishment among rural healthcare workers and lower rates of post-traumatic stress disorder and depersonalisation (rural 18.1%, metropolitan 20.7%). However, there was also evidence of increased negative effects among rural workers, such as higher levels of emotional exhaustion (rural 46.5%, metropolitan 43.3%; *P* = 0.002), mental health concerns (prevalent in 82%) and burnout levels. Another study, conducted early on in the COVID-19 pandemic, also reported that rural healthcare workers in China had higher rates of mental health difficulties, such as insomnia, anxiety and depression, although levels of burnout were not measured.^21^ Reasons given by the authors in this study included difficulties linked with rural locations and access to urgent and quality medical care. A more recent study from Romania showed no difference in burnout rates between rural and urban areas.^22^ Previous work has also suggested a higher rate of burnout among rural mental health providers, attributed to factors such as limited resources, geographical isolation, difficulties with recruitment and retention, and a tendency for rural dwellers to delay help-seeking and present later in their course of illness.^23^

The reverse occurred in our study, with higher rates of burnout being present among urban providers. Some researchers have, in fact, suggested a predilection for urban workers to have higher burnout rates. A pre-COVID-19 study examining burnout of family doctors working in rural and urban areas, found that urban-based doctors reported higher rates of burnout.^24^ The authors proposed several reasons for this finding: increased autonomy in rural settings, better relationships with patients, patients being more appreciative of their care and better personal and family lifestyle outside of the work.^24^ Other pre-COVID-19 studies suggested that in large cities, income is lower in relation to higher living costs, increased traffic and increased competition in the field of activity, and that this may explain those differences in medical personnel burnout between rural and urban areas.^22,25^ Although the lack of resources and increased case-load in rural and semi-rural areas have been hypothesised as risk factors for burnout, the present study and findings from previous research do not support this. Other factors previously reported in the literatures need to be investigated as possible protective factors for burnout. Health policies cannot explain this finding, as all MHS in our sample operate under the same policies. Perhaps lower levels of fear of contamination and lower prevalence rates of COVID-19 in rural and semi-rural areas compared with urban areas, lower rates of mental health referrals or mental health psychopathology among rural dwellers, and reduced impact of restrictions in less built-up areas are possible explanations, but those factors were not examined in this study.

Female gender was an independent predictor for both personal and work-related burnout. Previous studies have shown similar results across different settings and disciplines.^6,26^ However, not all studies have found this difference between genders. It was assumed that burnout is a female experience,^27^ but burnout may be experienced differently by men and women.^28^ Regional variation has been reported in the relationship between gender and burnout across countries, with higher rates found for women in USA studies, lower rates in European studies and no difference in Eastern Mediterranean and Arab countries, although the confound of lower female representation in the working population needs to be considered in certain countries.^29^ The scales used to measure burnout can influence the results (total burnout versus subscale burnout). For instance, in our sample when the total burnout scale was used in bivariate statistics, there was a significant difference between men and women, but this difference only holds for work-related and personal burnout, not patient-related burnout. Similarly, with the Maslach Burnout Inventory (MBI), a meta-analysis^30^ showed that women were more likely to be more emotionally exhausted than men (one subscale of the MBI); however, examining a different subscale of the MBI, men were more likely to report depersonalisation than women. The relationship between burnout and gender is complex, and statistical analyses, gender stereotypes, perception of women in their role in the workforce, and unrecorded ‘invisible’ care either in work or at home may explain this relationship.^31^

This study found that younger age was a risk factor for higher personal burnout and aligns with many previous studies reporting similar results.^6,26^ In a systematic review and meta-analysis of burnout in mental health professionals, O'Connor et al^16^ reported that levels of burnout typically decrease with age, whereas a study of neurologists suggested that the relationship between age and burnout may be non-linear, with a tendency to increase up to 40 years of age and then decrease.^32^ Our study found that age is related to personal burnout only, and may reflect personal choices in professional development and personal commitments outside of work, including family and social factors.

### Effort–reward imbalance and overcommitment

More effort with less reward (higher log-ERIdx) was an independent predictor for higher personal and work-related burnout, but was unrelated to patient-related burnout. Higher levels of overcommitment were associated with higher burnout across the three subscales. Similar results have been reported in previous studies of health professionals in Ireland^2,4^ and elsewhere, and in diverse occupations.^33–35^ Commitment to one's job has been viewed as a potentially protective position against adverse effects of stress, facilitating personal meaning and resolve to one's work, despite organisational difficulties.^36^ More recent work has highlighted the moderating role of the patient–doctor relationship. In a study by Moreno-Jiménez et al^37^ examining Spanish doctors, higher levels of commitment were associated with higher levels of turnover intentions in the presence of high frequency of difficult doctor–patient relationships, and may be explanatory in our findings of a positive relationship between overcommitment and burnout, including high patient-related burnout.

Although effort–reward imbalance and overcommitment are established risk factors for burnout, research has also reported the deleterious effects of both on physical and mental health. The theoretical foundations of the effort–reward imbalance model indicate high effort–low reward increases the possibility of negative emotions and sustained stress responses, whereas overcommitment increases risk of poorer mental and physical health.^12^ High scores on the ERI and overcommitment have been associated with increased risk of coronary heart disease^38^ and unhealthy behaviours, including smoking, alcohol and drug use.^39^ This study found no significant difference between clinical and non-clinical staff in any elements of the ERI, indicating similar risks across area of work within the organisation. Although clinical and non-clinical staff have different roles within the organisation, they all contribute to service provision. It is noteworthy that overcommitment represents a personal characteristic, whereas effort and reward reflect organisational/situational-specific components.^13^ This may explain the effect of overcommitment across all burnout domains, whereas effort–reward has an effect on personal and work-related burnout domains only. Our study findings might suggest (when considered alongside the evidence of low rates of patient-related burnout in relation to high personal and work-related burnout) that the contribution of organisational problems or ‘cultural’ attitudes within an organisation may contribute more significantly to burnout than internal factors or ‘vulnerability’ of the workers.

Leiter and Maslach,^40^ in an earlier seminal work, describe the importance of a ‘fit’ or ‘match’ between an organisation and an individual along six main work–life areas (workload, control, rewards, community, fairness and values), and reflect the major organisational antecedents to burnout. They argue of the importance for the ‘worker’ to perceived equity and fairness in reward received for effort given. Considering these areas in the context of mental health workers and the COVID-19 pandemic, staff workload increased, pandemic-induced uncertainty and loss of control were commonplace, and support from the community became less available with shift to working from home and reduced face-to-face contact. There may have been conflict between government/Health Service Executive and psychiatry clinical values in terms of containing the virus and prioritising physical well-being at the expense of psychological health and quality of life, with the associated increase in psychological distress and mental illness. There was a perception of lack of fairness and proportionality of restrictions applied having had disproportionate effects on vulnerable groups, such as those with mental health difficulties, those with intellectual disabilities, the elderly and the younger population. Mental health professionals may have felt that additional resources, be they staff or PPE, might not have been fairly applied, and those in the community not favourably rewarded or recognised for their effort. All these organisational factors need to be considered when examining burnout among staff, and in planning interventions that are appropriate and effective.^40^

### Limitations

One important limitation of the study is the difficulty in accurately establishing a response rate, given the uncertainty as to the number who opened an email invite; however, the low response rate here is consistent with response rates from other studies using similar recruitment methods.^6^ A second limitation is the selection of three MHS and lack of randomisation, thus limiting generalisability of the results; therefore the results cannot represent all workers in MHS in Ireland. Furthermore, the study was contacted in Western settings and this also limits the generalisability of the results. Finally, because this study was cross-sectional, causal and temporal relationship between burnout and practice environment and other demographic factors could not be determined. The strengths of this study include the use of validated scales, allowing for comparison with other studies, and the inclusion of non-clinical staff of the MHS who are excluded from most of the existing research, which tends to focus mainly on one discipline, usually physicians or nurses.

This research found a high rate of burnout, affecting 64.1% participants. Risk factors for burnout included female gender, younger age, urban service, time outside main responsibilities, overcommitment, high effort–reward imbalance, intention to change job and workload during the COVID-19 pandemic. Deterioration of personal mental and physical health during the pandemic and lack of opportunity to talk about work-related stress were also associated with higher burnout scores. These findings suggest that it is important to ensure the availability of support and that it is accessible to those at highest risk, including female and younger staff. Organisations have a responsibility to consider how best to support staff and to mitigate the effects of high workloads in times of crisis. There is a need for further research into organisational solutions to burnout, to sustain and build a healthy thriving workforce.

In conclusion, this study found high levels of personal and work-related burnout among mental health workers, but low levels of patient-related burnout. However, overcommitment was linked to higher degrees of patient-related burnout in additional to personal and work-related burnout, and highlights the importance of recognising potential adverse effects of strong personal commitment. Few variables directly relating to the COVID-19 pandemic were identified as risk factors for burnout. Although this may be partly explained by the timing of this study, conducted in the later stages of the pandemic, it also suggests the existence of burnout pre-pandemic, which was exacerbated by the additional strain placed on organisations and individuals by the COVID-19 pandemic. By mapping our findings to Leiter and Maslach's^40^ interconnecting domains of work–life, our findings highlight the importance of recognising seminal organisational factors that contribute to employee burnout. In addition, this study suggests that organisational factors (in this case Health Service Executive) may contribute more to burnout than internal factors of the workers, and those factors pre-dated the COVID-19 pandemic and perhaps will continue to exist.

## Data Availability

The data that support the findings of this study are available from the corresponding author, D.A., upon reasonable request.
